# A critical scientific evaluation of a purportedly negative data report – response to Seneviratne et al. 2022

**DOI:** 10.3389/fpsyt.2023.1271229

**Published:** 2023-10-04

**Authors:** Bankole Johnson, Giovanni Addolorato, Otto Lesch, Lei Liu, Zachary A. Rodd

**Affiliations:** ^1^Adial Pharmaceuticals, Inc., Charlottesville, VA, United States; ^2^Internal Medicine and Alcohol Related Disease Unit, Department of Medical and Surgical Sciences, Columbus-Gemelli Hospital, Fondazione Policlinico Universitario A. Gemelli IRCCS, Catholic University of Rome, Rome, Italy; ^3^CEMAD Digestive Disease Center, Fondazione Policlinico Universitario “A. Gemelli” IRCCS, Catholic University of Rome, Rome, Italy; ^4^Medical University of Vienna, Vienna, Austria; ^5^Division of Biostatistics, Washington University in St. Louis, St. Louis, MO, United States

**Keywords:** alcohol treatment, false negative, genotype, precision medicine, ethics, ondansetron, serotonin, 5HT-3 receptor

## Abstract

A core principle in the pursuit of scientific knowledge is that science is self-correcting and that important results should be replicable. Hypotheses need to be reinforced, adjusted, or rejected when novel results are obtained. Replication of results confirms hypotheses and enhances their integration into scientific practice. In contrast, publication of substantiated and replicated negative findings (i.e., non-significant or opposite findings) can be the basis to reject erroneous hypotheses or develop alternative strategies for investigation. Replication is a problem in all research fields. The Psychology Reproductivity Project reported that only 36% of ‘highly influential’ published research in highly ranked journals were reproduced. Similar to positive data, negative data can be flawed. Errors in a negative data set can be based on methodology, statistics, conceptual defects, and flawed peer review. The peer review process has received progressive scrutiny. A large-scale review of the peer review process of manuscripts submitted to the British Medical Journal group indicated that the process could be characterized as inconsistent, inaccurate, and biased. Further analysis indicated that the peer process is easily manipulated, indicative of a failed system, is a major factor behind the lack of replication in science (acceptance of flawed manuscripts), suppresses opposing scientific evidence and views, and causes gaps in and lack of growth of science. Complicating the integrity of scientific publication is the role of Editors/Researchers. Ethical guidelines exist for major publishing houses about editorial ethics, behavior, and practice.

## Introduction

A core principle in the pursuit of scientific knowledge is that science is self-correcting and that important results should be replicable. Hypotheses need to be reinforced, adjusted, or rejected when novel results are obtained (1). Replication of results confirms hypotheses and enhances their integration into scientific practice. In contrast, publication of substantiated and replicated negative findings (i.e., non-significant or opposite findings) can be the basis to reject erroneous hypotheses or develop alternative strategies for investigation.

Replication is a problem in all research fields. The Psychology Reproductivity Project reported that only 36% of ‘highly influential’ published research in highly ranked journals were reproduced (2). Similarly, only 11% of pre-clinical cancer studies were successfully replicated in multiple research laboratories (3). Despite the knowledge of a ‘replication crisis’ in science, the proportion of scientific manuscripts that declare support for the examined hypothesis has increased in recent years by 22%, while the publication of negative data is at a nadir (4). Similar to positive data, negative data can be flawed. Errors in a negative data set can be based on methodology, statistics, conceptual defects, and flawed peer review (5). The peer review process has received progressive scrutiny. A large-scale review of the peer review process of manuscripts submitted to the British Medical Journal (BMJ) group indicated that the process could be characterized as inconsistent, inaccurate, and biased (6). Further analysis indicated that the peer process is easily manipulated, indicative of a failed system, is a major factor behind the lack of replication in science (acceptance of flawed manuscripts), suppresses opposing scientific evidence and views, and causes gaps in and lack of growth of science (7).

Complicating the integrity of scientific publication is the role of Editors/Researchers. Ethical guidelines exist for major publishing houses about editorial ethics, behavior, and practice.^1^ Editors are typically allowed to publish in all journals, but there are specific recommendations/rules when there are competing interests. In cases of publishing in the journal in which an individual serves as an Editor, peer review should be performed blind to authors, and there should be a clear statement of the process published as a component of all manuscripts.

Because the publication of ‘false-negative’ data has a greater impact on scientific development than ‘true-positive’ and ‘false-positive’ data sets (8) great care needs to be applied in their publication. Basically, the introduction of ‘false-negative’ data to the scientific field can terminate the development of accurate pharmacological treatment of a disease, prevent additional researchers from obtaining funding and impair the replication of ‘true-positive’ research, and create an unwarranted bias against a legitimate treatment for a disease (9). Overall, reported ‘false-negative’ data can create an illegitimate threshold that can never be overcome by repeated ‘true-positive’ findings (8, 9).

The disproportionate effect of ‘false-negative’ data forced the creation of standards for the publication of negative results. The Elsevier Publishing Company has requirements for the publication of negative data.^2^ Key obligations include: (1) the data must be generated from correctly conducted research, (2) the negative results must be repeated multiple times by multiple collaborators to prevent ‘false-negatives’; (3) complete access must be provided to all methodological records of the experiment; (4) open access to raw data, and (5) allow for published response(s) to the negative data.

A recent publication in the official journal of the Research Society on Alcoholism reported negative findings for the use of ondansetron for the pharmacogenetic treatment of alcohol use disorder (AUD; (10); *Alcoholism: Clinical and Experimental Research*, 46, 1900–1912). The current commentary will discuss if the standards of publication of negative results were adhered to in that report and review concerns associated with the manuscript. The authors of this paper acknowledge a potential conflict interest and have disclosed these conflicts for consideration as to the merits of their comments in this manuscript. Nevertheless, the authors hold themselves to the highest scientific standards of transparency and integrity, and believe that it was important for the advancement of science to publish this manuscript.

### Preclinical support for the use of 5-HT 3 antagonists for the treatment of AUD

The evidence that the 5-HT 3 receptor mediates the actions of alcohol and alcohol consummatory behaviors is extensive. 5-HT 3 receptors located on ventral tegmental area (VTA) dopamine neurons are directly activated by alcohol and stimulate dopamine release in downstream projection areas (11). The rewarding actions of alcohol are thought to be, in part, regulated by the stimulation of dopamine release in the meso-cortico-limbic dopamine reward pathway (12). Antagonism of 5-HT 3 receptors in the VTA blocks the acquisition of alcohol self-administration, reduces on-going alcohol self-administration, and prevents enhanced alcohol relapse consumption following a period of deprivation (12). Recent data have indicated that activation of serotonergic neurons in the dorsal raphe, which projects to the posterior VTA and the nucleus accumbens shell (stimulating the 5-HT 3 receptor), regulate cue- and context-induced alcohol craving (11).

### Clinical support for the use of 5-HT 3 antagonists for the treatment of AUD

Preclinical research has translated into encouraging clinical trials. In healthy male volunteers, ondansetron, an antagonist of 5-HT 3 receptors, reduced the desire to drink alcohol and alcohol-induced mood changes (13). In an initial double-blind, randomized, placebo-controlled clinical trial (RCT), low-dose ondansetron showed promise to reduce alcohol consumption in heavy alcohol drinkers who consumed <10 drinks/drinking day (14). Ondansetron also has been shown to reduce alcohol consumption and increased abstinence in early-onset, but not late-onset, AUD patients (15). Treatment of AUD with ondansetron, categorized by clinical evidence identifying genetic polymorphisms and alcohol consumption endophenotypes (heavy alcohol drinking) was associated with the efficacy to decrease heavy drinking (16, 17). The senior author of the Seneviratne et al. (10) manuscript published positive clinical results of ondansetron (18). Furthermore, there is a positive phase 3 multi-center multi-national study of ondansetron for the precision medicine treatment of heavy drinking individuals with AUD in review with another journal. Thus, ondansetron can be considered the first precision medicine developed for the treatment of AUD, with replicated multi-site clinical trial findings.

## Discussion

### Seneviratne et al. (2022) results

There were 95 subjects enrolled in the two-site RCT. Subjects included individuals who were genetically responsive to ondansetron (*n* = 73) or genetically neutral (*n* = 22). The selected clinical endpoints were Drinks Per Drinking Days (DPDD, primary endpoint), Heavy Drinking Days (HDD), Drinking Days, and No Heavy Drinking Days (NHDD). The effect of ‘treatment site’ was the closest variable examined to significance (*p* = 0.08). Overall, the results indicated no effect of ondansetron on DPDD (*p* = 0.26), HDD (*p* = 0.12), DD (*p* = 0.59), or NHDD (*p* = 0.22).

### Critique 1: failure to replicate negative results

Six clinical studies have supported the efficacy of ondansetron in reducing alcohol consumption in AUD patients (c.f., (17)). Therefore, the need to replicate negative results should have been a requirement prior to the dissemination of the Seneviratne et al. (10) findings. In fact, the two sites in the study produced opposing results. For DPDD, 0.33 mg twice daily ondansetron reduced DPDD values (non-significantly) in the University of Maryland Baltimore (UMB) site. In contrast, at the University of Pennsylvania (UPenn) testing site, ondansetron non-significantly increased DPDD. Thus, internally, Seneviratne et al. (10) failed to replicate the non-significant effect of ondansetron on alcohol consumption. The study results for the UPenn testing site should have been examined for methodological issues (e.g., verification of ondansetron integrity) and replicated in a third location prior to the dissemination of the results. Given the small, under-powered sample size (*n* = 26 and 28 study completers, ondansetron and placebo, respectively), the researchers should have extended the RCT to a third site to prevent the publication of a detrimental conclusion ‘We found no evidence that low-dose oral ondansetron is beneficial in the treatment of AUD, irrespective of genotype, thus failing to confirm prior study findings’ (Abstract Conclusion, (10)). To restate, the Seneviratne et al. (10) report failed to replicate previous positive clinical data published by the senior author (18), which should have resulted in excessive scrutiny of the anomalous UPenn data.

### Critique 2: failure to select proper subgroup of AUD individuals for testing – genotype

*Post-hoc* genotype analysis of the Phase 2 clinical trial revealed 5 specific SNPs associated with the efficacy of ondansetron to reduce alcohol consumption in AUD subjects of European descent; the TT and LL variant of the serotonin transport (5-HTTLPR) and genotypic variations in the 5-HT 3A receptor (i.e., rs1150226-AG or rs1176713-GG) and 5-HT 3B receptor (i.e., rs17614942-AC; (16)). Furthermore, the genetic analysis indicated that the LL/TT variants needed to be co-expressed for ondansetron efficacy (16). The Seneviratne et al. (10) failed to require the combination of the LL/TT SNPs, introduced African American (A/A) subjects into the analysis with unsubstantiated genotypes (a missense polymorphism—rs1176744 in *HTR3B*), an unsubstantiated SNP for the 5-HTTLPR (rs25531:AA), and combinations of unsubstantiated SNPs with only the LL variant. In African American (A/A) subjects, Seneviratne et al. (10) did not include three 5-HT 3 receptor genotypes linked to the efficacy of ondansetron to reduce alcohol consumption in AUD subjects (AG, AC, and GG phenotype). The UPenn site had 80% (28 subjects) of the A/A subjects. Overall, the Seneviratne et al. (10) study did not use the same genotype as established in Johnson et al. (16) and included unsubstantiated genotypes in their ‘responsive’ condition. The failure to replicate repeated RCT indicating ondansetron was effective in reducing alcohol consumption in AUD patients could solely be based on the failure to adhere to established, correct genotypic classification (16).

### Critique 3: failure to select proper subgroup of AUD individuals for testing – alcohol consumption endophenotype

The refinement of ondansetron for the treatment of AUD is not limited to genotypic polymorphisms. Several studies have indicated that ondansetron is efficacious at reducing alcohol consumption in AUD subjects who consume <10 Standard Drinks/Drinking Days (<10 DDD). Very heavy drinkers (≥10 DDD) have endophenotypes associated with Binge Alcohol consumption and a reduction in the efficacy of treatments for AUD (19). RCT results have indicated that ondansetron is effective at reducing alcohol consumption mainly in heavy, but not very heavy, alcohol drinkers (16, 17). Seneviratne et al. (10) did not stratify alcohol consumption into heavy and very heavy drinkers. In Table 1 of the manuscript, it is reported that the percentage of heavy drinking days was 74% (18.3 SD), indicating a high likelihood of very heavy drinkers. Failure to follow the established methodology reported in other RCTs that examined the efficacy of ondansetron for the treatment of AUD combined with a low statistical power (discussed below) invalidates the negative results obtained in the Seneviratne et al. (10) study.

**Table 1:** Differences in BBCET procedures between Seneviratne et al. (10) and Johnson et al. (20).

Procedures	Seneviratne et al. (10)	Johnson et al. (20)	Potential consequence of the difference
Training for BBCET supervisors	No detail is provided on formal training of supervisors. Only PEPCO(TM) LLC can provide training for supervisors, and there is no documentation of such training being done.	Supervisors were trained and administered a test in which they had to achieve an 80% pass rate for certification. Certificates were reviewed and re-certification took place at scheduled intervals.	No evidence that BBCET supervisors or administrators were adequately or consistently trained. This could lead to “drift” in the administration of BBCET, which would no longer be a stable platform against which to measure the adjunctive effects of the medication.
Taping of BBCET sessions	No detail is provided on the audio taping of BBCET sessions for verification that BBCET procedures were being followed.	All BBCET sessions were taped and a random selection of 10% of audio tapes were inspected by a certified BBCET administrator. BBCET administrators that did not meet the standards of BBCET were provided feedback, re-trained, or certification was revoked if deviation from protocol was determined. This ensured that BBCET was being delivered accurately and with high fidelity.	No evidence that BBCET was administered consistently. Administrators in the Seneviratne et al. (10) study appear to not have been monitored for correct BBCET administration. Inconsistently administered BBCET can inflate the placebo response, making it more difficult to detect any therapeutic effect of active treatment.
Manual for BBCET administration in accordance with the protocol	States that a manual was produced but this was not submitted with the supplementary materials of the publication, and no information on how to obtain the manual is provided. The manual is likely not certified by a qualified BBCET supervisor.	Manuals were provided for BBCET for all administrators and supervisors. All these manuals were standardized and readily available from the lead author. Manuals were certified by a qualified BBCET supervisor using established procedures, having been reviewed by a team of other supervisors.	Standardization of the BBCET manual is important for its consistent administration. Otherwise, it is uncertain what psycho-social treatment was actually administered.
Schedules for BBCET dependent on the time in study	A schedule of three patterns of BBCET focus is provided. Phase 1 is initiation, Phase 2 maintaining treatment adherence, and Phase 3 “how to maintain their desired level of drinking (abstinence or non harmful) after leaving the study”.	BBCET is delivered in focused patterns during the study. In contrast to its use in the study of Seneviratne et al. (10), the focus of BBCET, as a motivational and behavioral tool, was on the present. BBCET does not include a module for further prevention and is not a preventative tool. For behavioral tools to be effective, they must be linked with proximate real time events. The stated procedure of Seneviratne et al. (10), therefore, deviates from the principles of BBCET. The Seneviratne study deviates even further from the tenets of BBCET in its focus on directed drinking behavior. In BBCET, the client sets his or her own “target drinking” goal (i.e., it can be positive or negative in direction). This is not manipulated by the BBCET administrator. This is important because it links the behavior to actual consequences, which is critical for a well administered behavioral management strategy. Of course, if the participant repeatedly changes drinking targets that have negative consequences, there are procedures within BBCET to help with a correction but they are not directed.	The deviation from standard BBCET procedures introduces the element of clinical variation. In particular, the BBCET administrator directing the participant on expected desired drinking behavior can introduce potential bias by creating an artificial target platform. In standardly administered BBCET, the participant can even choose a drinking goal that is undesired (e.g., more drinking or less abstinence) and is, therefore, bidirectional. Not allowing a bidirectional consequence can prevent the participant from linking real time tools with behavioral consequences related to the target drinking goal. This limits the behavioral effect of BBCET to help the participant develop simple corrective behavioral tools associated with altering drinking behavior.
Procedures	Seneviratne et al. (8)	Johnson et al. (18)	Potential consequence of the difference
Educational component of BBCET	Participants were educated on the “use of medication to treat AUD” from weeks 4 - 10. BBCET also was used to educate on the consequences of excessive alcohol consumption. Medication compliance for both treatment groups averaged approx. 78–83%.	BBCET was also delivered to provide education on the consequences of excessive alcohol consumption and the potential effects and, importantly, the specific adverse events associated with that particular medication. This is critical to maintaining adherence to pill taking. Pill-taking compliance measures were started in the second study week, and emphasized throughout the study. The reported pill-taking rate was approx. 91% for both treatment and placebo groups.	Considering that the Johnson et al. (20) study was testing a different medication to ondansetron (i.e., topiramate) with a markedly higher frequency and severity of adverse events profile, the reported pill-taking rate in the Seneviratne et al. (10) study was relatively low. As an example, in the present recently completed 24-week Phase III study presented at ESBRA on September 1, 2023, using a similar dose of ondansetron to the 14- week Seneviratne et al. (10) study, the pill-taking rate for the active treatment was 95.6% and for placebo 98.8%. This underscores the proposal that the Seneviratne et al. (10) study did not appear to achieve the pill-taking compliance expected with standard BBCET administration.
Discussion of compliance barriers	Discussion of compliance barriers is stated as a tool in their administration of BBCET.	BBCET was not used to discuss compliance barriers. An important tenet of BBCET is not to discuss compliance barriers, which is more typical of cognitive behavioral therapy or formal psychotherapy. BBCET is not a psychotherapy and provides no information on the interpretation of behaviors or feelings.	Delivering alternate or additional other treatments within the BBCET framework limits its standardization to be applied to generic treatment practices. Also, it could have the effect to increase the placebo rate, which would reduce the ability to detect the effect of the active treatment.

### Critique 4: failure to report numerical values of AUD-associated variables

The ethics of conducting RCTs include publishing all the results of the clinical trial. Publishing negative results requires further complete disclosure of all record variables and methodological procedures (4, 5). In the Seneviratne et al. (10) manuscript, no specific numerical values are provided for any study variable in either of the two arms. Instead, the presented values represent the differences relative to the placebo group. With no reported values, there is no way to determine if the placebo groups were “normal” compared to other AUD RCTs. Furthermore, the Seneviratne et al. (10) trial was conducted for 4 months, and there was no presentation of the temporal values of the multiple clinical endpoints examined.

### Critique 5: inadequate statistical power

Accurate statistical analyses of RCT data require proper statistical power. The Seneviratne et al. (10) study had a NIH project start date of April 20, 2012 with a target recruitment number of 256; yet, the performance period was reported as 2015–2020, and the sample size of genetically responsive subjects was low (26 and 28 study completers, ondansetron and placebo, respectively). Although no numerical values were reported in the manuscript, the power estimate for the proposed experiment of Seneviratne et al. (10) was based upon the Johnson et al. (16) data set. Utilizing the effect size obtained in Johnson et al. (16), we calculated the power of Seneviratne et al. (10) at 0.20, which is insufficient to make any valid inferential comparisons (details regarding the power calculation can be found in the Supplementary material). Thus, the manuscript failed to adhere to another standard for the publication of negative results: data must be unequivocally statistically negative based on accurate and rigorous data analyses. In sum, the data was divergent based on RCT test site and statistically underpowered to the level that would prevent definitive conclusions.

### Critique 6: adherence to psychological treatment

Seneviratne et al. (10) attempted to replicate the findings of Johnson et al. (16) by utilizing the same psychological treatment. The Brief Behavioral Compliance Enhancement Treatment (BBCET) is a brief (15–30 min per person) standardized treatment platform (20). BBCET is performed in conjunction with pharmacological interventions for the treatment of AUD. BBCET is a sensitive tool and must be applied consistently to subjects to avoid inflation of placebo response which would ‘mask’ any putative therapeutic effects of the treatment (20). BBCET is a proprietary product delivered under copyright license (BA Johnson) and requires extensive training and supervision to be applied properly. Despite past concerns of intellectual property violations, Seneviratne et al. (10) failed to license BBCET or receive/provide standardized training for individuals to administer the test. Differences in training and application of BBCET between the testing sites of Seneviratne et al. (10) could be the basis for the divergence in the efficacy of ondansetron to reduce alcohol consumption.

### Negative reported RCT results for pharmacotherapeutics for the treatment of AUD

There are three US FDA-approved treatments for AUD: Antabuse, naltrexone, and acamprosate. In a large RCT, naltrexone failed to alter multiple measures of alcohol consumption in AUD subjects (21). Although eventually FDA-approved, naltrexone has a modest effect on alcohol consummatory behaviors. In the largest naltrexone clinical trial (COMBINE) in patients receiving only drug treatment (100 mg daily) for 4 months, there was a 5.5% treatment effect for the ‘increase in percentage of days of abstinence’ and a 6.9% treatment effect for the ‘reducing the risk of a heavy-drinking day’ (22). Numerous large-scale RCT have failed to observe significant effects of acamprosate on reducing alcohol consummatory behaviors/abstinence in AUD subjects [c.f., (23)]. Similarly, RCT results of nalmefene, the EMA-approved pharmacotherapeutic for AUD, have been characterized as suffering from bias, post-hoc sample refinement, inappropriate comparators, and (at best) modest or uncertain efficacy to individual patients (24). Unlike the negative RCT findings of other approved pharmacotherapies for the treatment of AUD, the Seneviratne et al. (10) manuscript has methodological and statistical flaws. The FDA and EMA could approve ondansetron in the future, but the potential damage of a single false-negative study report could have an impact on the willingness of clinicians to use the compound to treat selected AUD patients.

## Conclusion

There is a conflicting need in science. Publication of negative data is a requirement for the development of accurate scientific concepts (4). Yet, false-negative data can have a disproportionate effect on the development of potentially important pharmaceuticals (8). There are guidelines for the publication of negative data to assist in the dissemination of potentially highly influential (accurate and inaccurate) reports. The Research Society on Alcoholism and *Alcohol Clinical and Experimental* Research should develop standards for the publication of negative data. The Seneviratne et al. (10) manuscript fails to adhere to the guidelines for publication of negative data and we have found significant limitations in the data presented in that study. Currently, <10% of AUD patients are treated with pharmacological agents for their condition (25). Clinician belief that there is no efficacious or tolerated treatment for AUD mediates the low use rate of pharmacological treatment of AUD (25). Ondansetron has been shown to be efficacious in reducing alcohol consumption in a genetically-selected, consummatory subgroup (<10 DDD) of AUD individuals (16). It would be a detrimental consequence of the Seneviratne et al. (10) manuscript if further development of a well-tolerated precision medicine treatment for AUD is impaired.

## Data availability statement

The original contributions presented in the study are included in the article/Supplementary material, further inquiries can be directed to the corresponding author.

## Author contributions

BJ: Conceptualization, Project administration, Writing – original draft, Writing – review & editing. GA: Writing – review & editing. OL: Writing – review & editing. LL: Methodology, Writing – review & editing. ZR: Writing – review & editing.

## Footnotes

^1^ https://www.elsevier.com/about/policies/publishing-ethics

^2^ https://www.journals.elsevier.com/resources-conservation-and-recycling/news/negative-results-are-publishable
