# Supplementary material for: A critical scientific evaluation of a purportedly negative data report – response to Seneviratne et al. 2022
Source: Front Psychiatry. 2023 Oct 4;14:1271229. doi: 10.3389/fpsyt.2023.1271229 (PMC10582924; doi:10.3389/fpsyt.2023.1271229)
Supplement: Supplementary file 1 [file Data_Sheet_1.PDF]

SUPPLEMENTAL STATISTICAL INFORMATION

We carried out a power analysis employing the bootstrapping technique as detailed by Efron (1979), which leverages random sampling with replacement to replicate the real data distribution. We opted for nonparametric bootstrapping, thus refraining from making any parametric assumptions about the data's structure. Given our dataset's longitudinal nature, we turned to the cluster bootstrapping approach, i.e., we sampled entire subjects rather than individual data points within them, as suggested by Field and Welsh (2007). Our implementation aligns with Hyslop (1995), executed in SAS. For the sample size specified in Seneviratne et al. (2022), we generated 1000 bootstrapped datasets. Subsequently, we utilized the mixed model for repeated measures (MMRM) to evaluate these bootstrapped sets. The power is calculated as the proportion of significant outcomes amongst the 1000 bootstrapped datasets.

References

Efron, B. (1979). Bootstrap methods: Another look at the jackknife. *The Annals of Statistics*. **7** (1): 1–26

Field, C.A. and Welsh, A.H. (2007), Bootstrapping clustered data. *Journal of the Royal Statistical Society: Series B (Statistical Methodology)*, **69**: 369-390.

Hyslop T. (1995). SAS Macros for Bootstrap Samples with Stratification and Multiple Observations per Subject. URL: www.lexjansen.com/nesug/nesug95/NESUG95148.pdf
